# GRIK phosphorylates and activates KIN10 which also promotes its degradation

**DOI:** 10.3389/fpls.2024.1375471

**Published:** 2024-03-25

**Authors:** Jing Sun, Hui Liu, Jantana K. Blanford, Yingqi Cai, Zhiyang Zhai, John Shanklin

**Affiliations:** Biology Department, Brookhaven National Laboratory, Upton, NY, United States

**Keywords:** SnRK1, KIN10, GRIK1, protein kinase, alternative splicing isoform

## Abstract

The sensor kinase Sucrose Non-fermenting-1-Related Kinase 1 (SnRK1) plays a central role in energy and metabolic homeostasis. KIN10 is a major catalytic (α) kinase subunit of SnRK1 regulated by transcription, posttranslational modification, targeted protein degradation, and its subcellular localization. Geminivirus Rep Interacting Kinase 1 and 2 (GRIK1 and 2) are immediate upstream kinases of KIN10. In the transient protein expression assays carried out in *Nicotiana benthamiana (N. benthamiana)* leaves, GRIK1 not only phosphorylates KIN10 but also simultaneously initiates its degradation. Posttranslational GRIK-mediated KIN10 degradation is dependent on both GRIK kinase activity and phosphorylation of the KIN10 T-loop. KIN10 proteins are significantly enriched in the *grik1-1 grik2-1* double mutant, consistent with the transient assays in N. benthamiana. Interestingly. Among the enriched KIN10 proteins from *grik1-1 grik2-1*, is a longer isoform, putatively derived by alternative splicing which is barely detectable in wild-type plants. The reduced stability of KIN10 upon phosphorylation and activation by GRIK represents a mechanism that enables the KIN10 activity to be rapidly reduced when the levels of intracellular sugar/energy are restored to their set point, representing an important homeostatic control that prevents a metabolic overreaction to low-sugar conditions. Since GRIKs are activating kinases of KIN10, KIN10s in the *grik1 grik2* double null mutant background remain un-phosphorylated, with only their basal level of activity, are more stable, and therefore increase in abundance, which also explains the longer isoform KIN10L which is a minor isoform in wild type is clearly detected in the *grik1 grik2* double mutant.

## Introduction

Plant Sucrose Non-fermenting-1-Related Kinase 1 (SnRK1) belongs to a family of Ca^2+^-independent serine/threonine protein kinases that are related to the Sucrose Non-Fermenting 1 (SNF1) kinase found in fungi, and the AMP-activated protein kinase (AMPK) in animals (Broeckx et al., 2016). In plants, SnRK1 functions as an important metabolic sensor kinase that is activated under low carbon/energy conditions. Activated SnRK1 phosphorylates a constellation of target proteins including key transcription factors and metabolic enzymes that results in a broad reprogramming of metabolism (Sugden et al., 1999; Baena-González et al., 2007; Tsai and Gazzarrini, 2012; Mair et al., 2015; Zhai et al., 2017). SnRK1 is a heterotrimeric complex composed of a catalytic α subunit (encoded by *KIN10 and KIN11*, also known as *SnRK1α1*, and *SnRK1α2*, respectively) in Arabidopsis and regulatory subunits: β and βγ (Ramon et al., 2013; Emanuelle et al., 2015; Peixoto and Baena-González, 2022). KIN10 is also capable of activity independent of its regulatory subunits (Ramon et al., 2019). KIN10 is broadly expressed while KIN11 expression is restricted to specific tissues and developmental stages (Williams et al., 2014). Alternate splicing of KIN10 results in two KIN10 protein isoforms. The long KIN10 isoform (referred to as KIN10L herein) has a 23 residue N-terminal extension relative to the short KIN10 (referred to as KIN10). KIN10 appears to be the major KIN10 form *in planta* because the transcript levels of KIN10 in multiple tissues under laboratory growth conditions are reported to be much higher than those of KIN10L (Williams et al., 2014). In terms of physiological functions, overexpression of KIN10 leads to hypersensitivity to glucose and abscisic acid (ABA) (Jossier et al., 2009). It also increases leaf soluble sugar (i.e., glucose, Fructose, and sucrose) content (Jossier et al., 2009; Wang et al., 2019). Significant amounts of starch are detected in the *kin10 kin11* double mutant at the end of the dark period suggesting that KIN10 and KIN11 are involved in mobilizing starch during darkness (Baena-González et al., 2007). While the functions of KIN10 and KIN11 largely overlap, some differences have been noted, for instance, KIN10 overexpression delayed flowering while KIN11 overexpression promoted flowering (Baena-González et al., 2007; Tsai and Gazzarrini, 2012; Williams et al., 2014; Wang et al., 2019). KIN10 was reported to positively regulate stomatal development under high sucrose conditions. Both *kin10* and *kin11* single mutants showed lower stomatal index relative to wild type (Han et al., 2020). For a broader description of the functions of KIN10 and KIN11, please refer to the following reviews (Crepin and Rolland, 2019; Margalha et al., 2019; Baena-González and Lunn, 2020; Peixoto and Baena-González, 2022).

Two Arabidopsis SnRK1 activating kinases, SnAK1 and SnAK2, also widely referred to as GRIK2 and GRIK1(Geminivirus Rep Interacting Kinase 1 and 2) respectively, are upstream activating kinases of KIN10 and KIN11. They activate KIN10 or KIN11 by phosphorylating T175 in KIN10 activation loop (T-loop) or T176 in KIN11 T-loop. The phosphorylation activates their *in vitro* activity (Shen and Hanley-Bowdoin, 2006; Hey et al., 2007; Shen et al., 2009). Consequently, in the *grik1-1 grik2-1* double mutant, T-loop phosphorylation is not observed (Glab et al., 2017). The *grik1-1 grik2-1* double mutant displays a dwarfed growth habit and is infertile (Glab et al., 2017), phenocopying the *kin10 kin11* double mutant (Baena-González et al., 2007), consistent with essential *in vivo* roles of GRIKs in the activation of SnRK1. It has also been reported that activated KIN10 can phosphorylate and inhibit the activity of GRIKs *in vitro*, providing evidence for negative feedback regulation of KIN10 activation (Crozet et al., 2010).

The phosphorylated disaccharide trehalose 6-phosphate (T6P) acts as a signal of intracellular sucrose availability connecting plant growth and development to its metabolic status (Schluepmann et al., 2003; Lunn et al., 2006; Yadav et al., 2014; Figueroa and Lunn, 2016). We recently reported that at physiologically relevant levels, T6P directly binds to KIN10 weakening the affinity of GRIK1 for KIN10, thereby reducing the phosphorylation of KIN10’s T-loop, KIN10 activation and SnRK1 activity (Zhai et al., 2018).

Besides activation of KIN10/11 by its upstream kinases, selective degradation of KIN10/11 is a mechanism that attenuates SnRK1 signaling and prevents detrimental hyperactivation during responses to stresses. KIN10 can interact with Pleiotropic Regulatory Locus 1 (PRL1) (Bhalerao et al., 1999) and KIN10 degradation is mediated by the DDB1-CUL4-ROC1-PRL1 E3 ubiquitin ligase, via its interaction with the KIN10-PRL1 complex (Lee et al., 2008). Under low-nutrient conditions, myoinositol polyphosphate 5-phosphatase 13 (5PTase13) is required to stabilize KIN10 and slow its degradation by the 26S proteasomal pathway (Ananieva et al., 2008). It has been demonstrated that application of ABA to wheat roots can result in a dramatic reduction of KIN10 (Coello et al., 2012). KIN10 degradation is reported to be strictly dependent on its kinase activity because two KIN10 kinase mutants: T175A and K48M (impaired in their phosphotransferase activity) accumulate to higher levels than wild type KIN10 due to its reduced degradation (Baena-González et al., 2007; Crozet et al., 2016). In other studies, KIN10/11 were also found to be SUMOylated by SIZ1 (E3 Small Ubiquitin-like Modifier (SUMO) ligase), marking them for proteasomal degradation (Crozet et al., 2016).

Based on the observations that GRIK is the major kinase that phosphorylates and activates KIN10 at its T-loop and that a KIN10 T-loop mutant [KIN10 (T175A)] shows increased stability relative to KIN10, we tested whether GRIK is directly involved in KIN10 degradation.

Here, we report that transient co-expression of KIN10 with GRIK1 in *Nicotiana benthamiana* (*N*. *benthamiana*) leaves results in significant degradation of KIN10 and that the GRIK1-dependent KIN10 degradation is contingent on the kinase activity of GRIK1 in phosphorylating the KIN10 T-loop.

Consistently, KIN10 protein levels are significantly elevated in the *grik1-1 grik2-1* double mutant. Two isoforms of KIN10 are identified upon immunoprecipitations using KIN10 antibody from the *grik1-1 grik2-1* double mutant, among them is a long alternative splicing isoform that is a minor isoform in wild-type plant.

## Results

### GRIK1 phosphorylates KIN10 promoting its degradation

**Figure 1:**
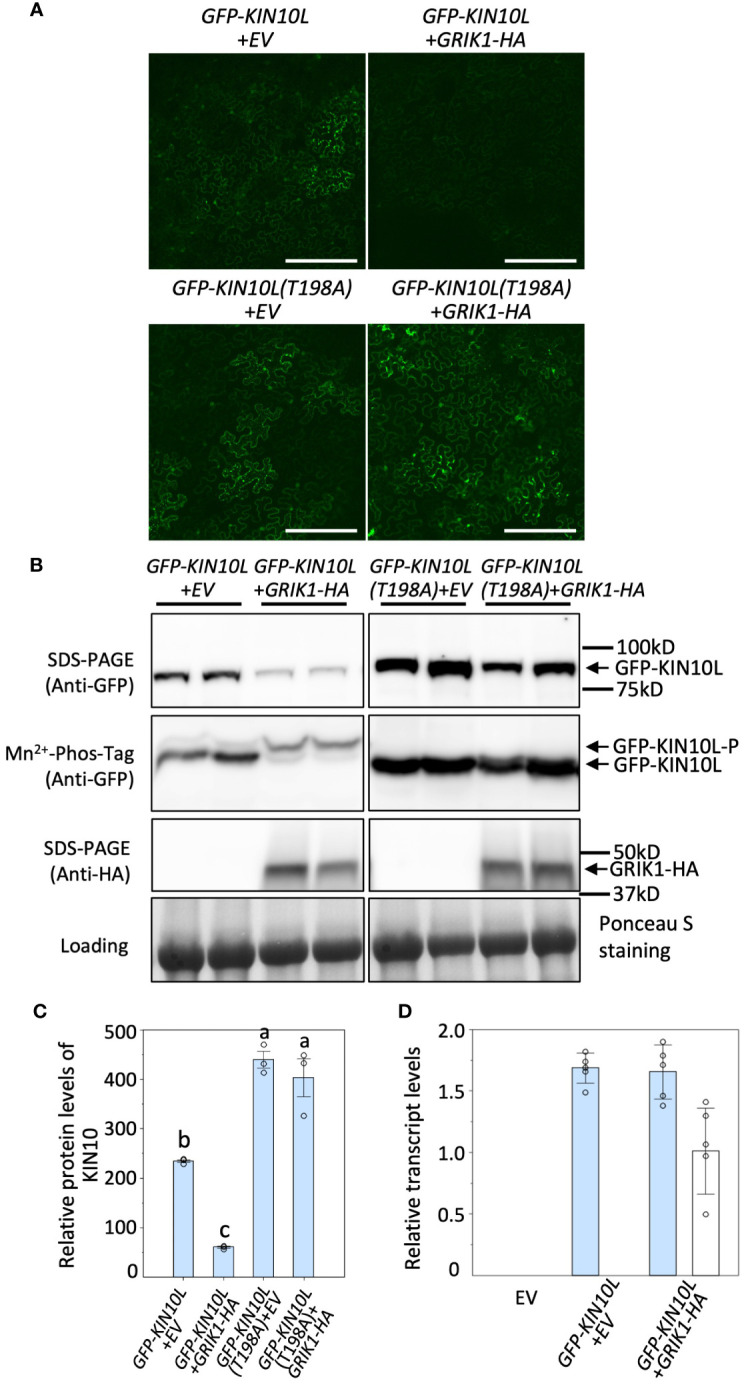
Overexpression of *GRIK1* results in KIN10 degradation in *N. benthamiana* Leaves. **(A)** Representative fluorescence confocal images of *N*. *benthamiana* leaf samples 3 d after co-agroinfiltration with gene expression combinations as shown. EV, empty vector. KIN10L, long splicing protein isoform of *KIN10*. KIN10L(T198A), a KIN10L mutant. Bar = 250 mm. **(B)** immunoblot analysis of samples in **(A)** shows protein levels of total GFP-KIN10L or GRIK1-HA and respective phosphorylated (GFP-KIN10L-P) and non-phosphorylated GFP-KIN10L (Mn^2+^-Phos-tag is a 10% SDS-PAGE containing 50μM of Mn^2+^-Phos-tag™. Ponceau S staining of Rubisco is shown as a loading control. In all figures, multiple lanes for one gene combination or one genotype represent biological replicates. **(C)** Relative GFP-KIN10L protein levels in **(B)** quantified with GelAnalyzer2010 and normalized against corresponding protein loading. Data shown are mean ± SD, *n*=3 independent immunoblots; One-way analysis of variance (ANOVA) and Tukey-Kramer Honestly Significant Difference (P <0.05) are used to compare means. Different letters above boxes indicate a significant difference. **(D)** Reverse transcription quantitative PCR (RT-qPCR) results of *KIN10L* and *GRIK1* in **(A)**, values are means ± SD, *n*=5 independent experiments. Statistics is performed by using mean crossing point deviation analysis computed by the relative expression [REST] software algorithm. The blue bars represents gene transcript for *GFP-KIN10L* and the open bars represents for gene transcript for *GRIK1-HA*.

It was previously reported that KIN10 degradation is strictly dependent on its kinase activity (Baena-González et al., 2007; Crozet et al., 2016). Since GRIK is the major kinase that phosphorylates and activates KIN10, we tested whether GRIK is involved in KIN10 degradation. GFP signal from GFP-tagged KIN10L was monitored upon transient co-expression of KIN10L with GRIK1 in *Nicotiana benthamiana* (*N*. *benthamiana*) leaves by fluorescence microscopy (**Figure 1A**) and western blotting with anti-GFP antibodies (**Figure 1B**). Previously, it was shown that Threonine-198 (T198) in the T-loop of KIN10L (equivalent to T175 in the T-loop of KIN10) is phosphorylated by GRIK and essential for KIN10 kinase activity (Shen et al., 2009). Consistent with previous reports, expression of the KIN10L T198A phosphorylation mutant resulted in increased protein accumulation relative to KIN10L (**Figures 1A–C**). Co-expression of GRIK1 with KIN10L greatly reduced KIN10L accumulation relative to the expression of KIN10L alone (**Figures 1A–C**). Mn^2+^-Phos-tag gel electrophoresis was used to separate phosphorylated from non-phosphorylated proteins, revealed that most of the residual KIN10L upon its co-transformation with GRIK1 was present in phosphorylated form (**Figure 1B**). There was only a single detected band for GFP-KIN10L (T198A) visible in the Mn^2+^-Phos-tag gel blot upon co-transformation with GRIK1-HA (**Figure 1B**), suggesting that T198 in the T-loop is the only phosphorylation site for GRIK1. That the T-loop phosphorylation mutant of KIN10L was strongly stabilized relative to its parental wild-type sequence also suggests posttranslational regulation. To confirm this, we compared the levels of GFP-KIN10L mRNA upon its expression alone versus upon its co-expression with GRIK1-HA. The levels of GFP-KIN10L transcripts were equivalent for both treatments (**Figure 1D**), confirming that the observed reduction in GFP-KIN10 protein occurs at the posttranslational level.

**Figure 2:**
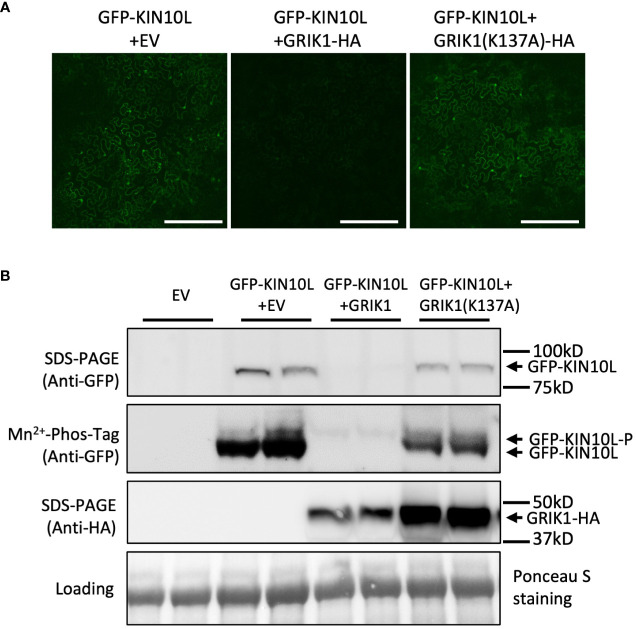
GRIK1 mediated KIN10 degradation is dependent on GRIK1 kinase activity. **(A)** Representative fluorescence confocal images of *N*. *benthamiana* leaf samples 3 d after co-agroinfiltration with gene expression combinations as shown. GRIK1(K137A) and GRIK1 (S261A) are GRIK1 mutants. Bar = 250 mm. **(B)** immunoblot analysis of samples in **(A)** shows protein levels of total GFP-KIN10 or GRIK1-HA and respective phosphorylated (GFP-KIN10-P) and non-phosphorylated GFP-KIN10 (Mn^2+^-Phos-tag). Ponceau S staining of Rubisco is shown as a loading control.

To further understand GRIK-mediated KIN10 degradation we engineered a GRIK1 mutant, K137A. K137 is a key residue in the ATP binding domain of GRIK1 reported to be essential for its kinase activity (Shen et al., 2009). Co-transformation of KIN10 with GRIK1(K137A) did not result in substantial KIN10 degradation, confirming that GRIK1-mediated KIN10L degradation is dependent on the kinase activity of GRIK1 (**Figures 1A, B**, **2A, B**).

### The KIN10 long splicing isoform is enriched in a *grik* null mutant

**Figure 3:**
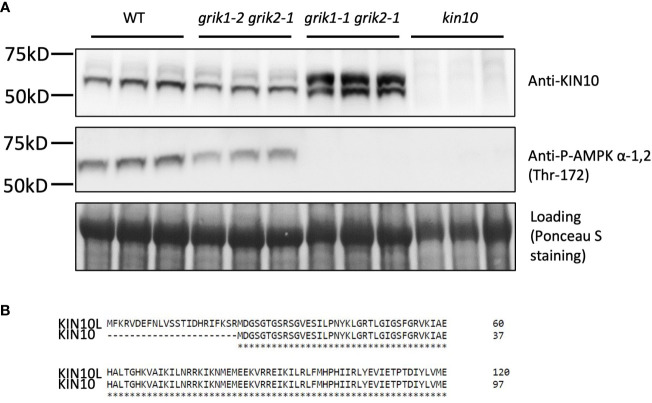
KIN10 protein levels are significantly higher in the *grik1-1grik2-1* double knockout mutant seedlings than that in wild type Arabidopsis. **(A)** immunoblot analysis of proteins extracted from 10-day-old of seedlings of WT, *grik1-2grik2-1* (a weak *grik1grik2* double mutant), *grik1-1grik2-1* (a strong *grik1grik2* double mutant) and *kin10* respectively. KIN10 or P-AMPK α-1 (Thr-172) antibody was used to detect KIN10 or phosphorylated KIN10 respectively. Ponceau S staining of Rubisco is shown as a loading control. **(B)** Protein sequence alignment of long (KIN10L) and short (KIN10) alternative splicing isoforms of KIN10 shows 23 more amino acid residues on N terminus of KIN10L than KIN10.

To substantiate GRIK1-mediated KIN10 degradation we observed in transient *N*. *benthamiana* leaf assays, KIN10 protein levels were quantified in two Arabidopsis *grik* double mutants: *grik1-2 grik2-1* containing the weaker *grik1-2* allele and *grik1-1 grik2-1* containing the stronger *grik1-1* allele of *GRIK1*. Consistent with published results (Glab et al., 2017), phosphorylated KIN10 was observed in the *grik1-2 grik2-1* line, but not in *grik1-1 grik2-1*, as evidenced by probing with the phosphorylated KIN10-specific antibody. This confirms that GRIK1 and GRIK2 are major activating kinases of KIN10 *in vivo* (**Figure 3A**). Immunoblot assays probed with the KIN10-specific antibody showed that two distinct forms of KIN10 are detected in WT and the *grik* mutants but no KIN10 immunoreactive species are visible in the *kin10* mutant. The higher molecular mass KIN10 form was significantly more abundant in *grik1-1 grik2-1* than in either WT or *grik1-2 grik2-1* (**Figure 3A**). Since KIN10 has two alternative splicing isoforms (i.e., KIN10L and KIN10) (**Figure 3B**), we hypothesize that the large and small KIN10s detected in *grik1-1 grik2-1* are KIN10L and KIN10, respectively.

The individual coding sequence (CDS) corresponding to *KIN10L* or *KIN10* was transiently expressed in *N*. *benthamiana* leaves. Three days after agroinfiltration, protein samples were extracted and separated along with protein samples from the *grik1-1 grik2-1* double mutant and subjected to immunoblotting. Transiently expressed KIN10L and KIN10 polypeptides in *N*. *benthamiana* showed similar SDS-PAGE mobilities to those of the putative KIN10L and KIN10 products in *grik1-1 grik2-1* respectively (**Supplementary Figure 1**). To further confirm the identity of two sizes of KIN10 detected in the *grik1-1 grik2-1* double mutant, KIN10s were immunoprecipitated with anti-KIN10 antibody from *grik1-1 grik2-1* and separated by SDS-PAGE. The two protein bands were excised and analyzed with the use of tandem mass spectrometry. The faster migrating protein was confirmed to be KIN10. The slower migrating protein was identified as the KIN10L isoform (**Supplementary Figure 2**). These data show that KIN10L, a minor KIN10 isoform in WT, is enriched in the *grik1-1 grik2-1* double mutant background due to the increased protein stability of its unphosphorylated form in *grik1-1 grik2-1*.

### KIN10L accumulates to higher levels than KIN10 when transiently expressed in *N*. *benthamiana* leaves

**Figure 4:**
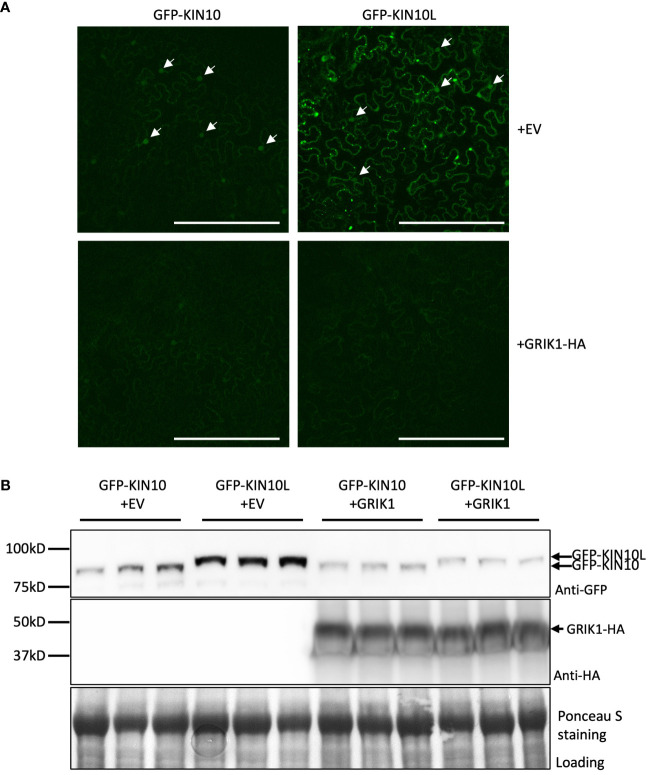
The long KIN10 isoform (KIN10L) accumulates to higher levels than KIN10 when transiently expressed in *N*. *benthamiana* leaves. **(A)** Representative fluorescence confocal images of *N*. *benthamiana* leaf samples 3 d after co-agroinfiltration with gene expression combinations as shown. Bar = 250 mm. Fluorescence signal in nucleus is marked by white arrowhead. **(B)** immunoblot analysis of samples in **(A)** shows protein levels of GFP-KIN10L or GFP-KIN10 or GRIK1-HA. Ponceau S staining of Rubisco is shown as a loading control.

To evaluate whether there are differences between KIN10L and KIN10, GFP-KIN10L or GFP-KIN10 were transiently co-expressed for 3 days in *N*. *benthamiana* leaves with either GRIK1 or an empty vector. As shown in **Figure 4**, compared with GFP-KIN10, more intense GFP fluorescence corresponding to GFP-KIN10L was observed as the puncta in the cytosol, consistent with reports by Williams (Williams et al., 2014) (**Figure 4A**). Immunoblot assays showed the levels of GFP-KIN10L were significantly higher than GFP-KIN10 (**Figure 4B**) upon co-expression with EV. Co-expression with GRIK1 dramatically reduced both KIN10L and KIN10 protein levels (**Figure 4B**). The accumulation of KIN10L seems related to its subcellular localization because a putative nuclear localization signal mutant of KIN10L (K250A, K251A, K253A) in the sequence LFKKIKG which is a match to monopartite nuclear localization signal K·(K/R) ·X·(K/R) (Chelsky et al., 1989), accumulated to higher levels than native KIN10L. Conversely, fusing KIN10L with the SV40 NLS resulted in almost complete retention of KIN10L within the nucleus and promoted its degradation (**Supplementary Figure 3**).

### Kinase activity of KIN10L is equivalent to that of KIN10

**Figure 5:**
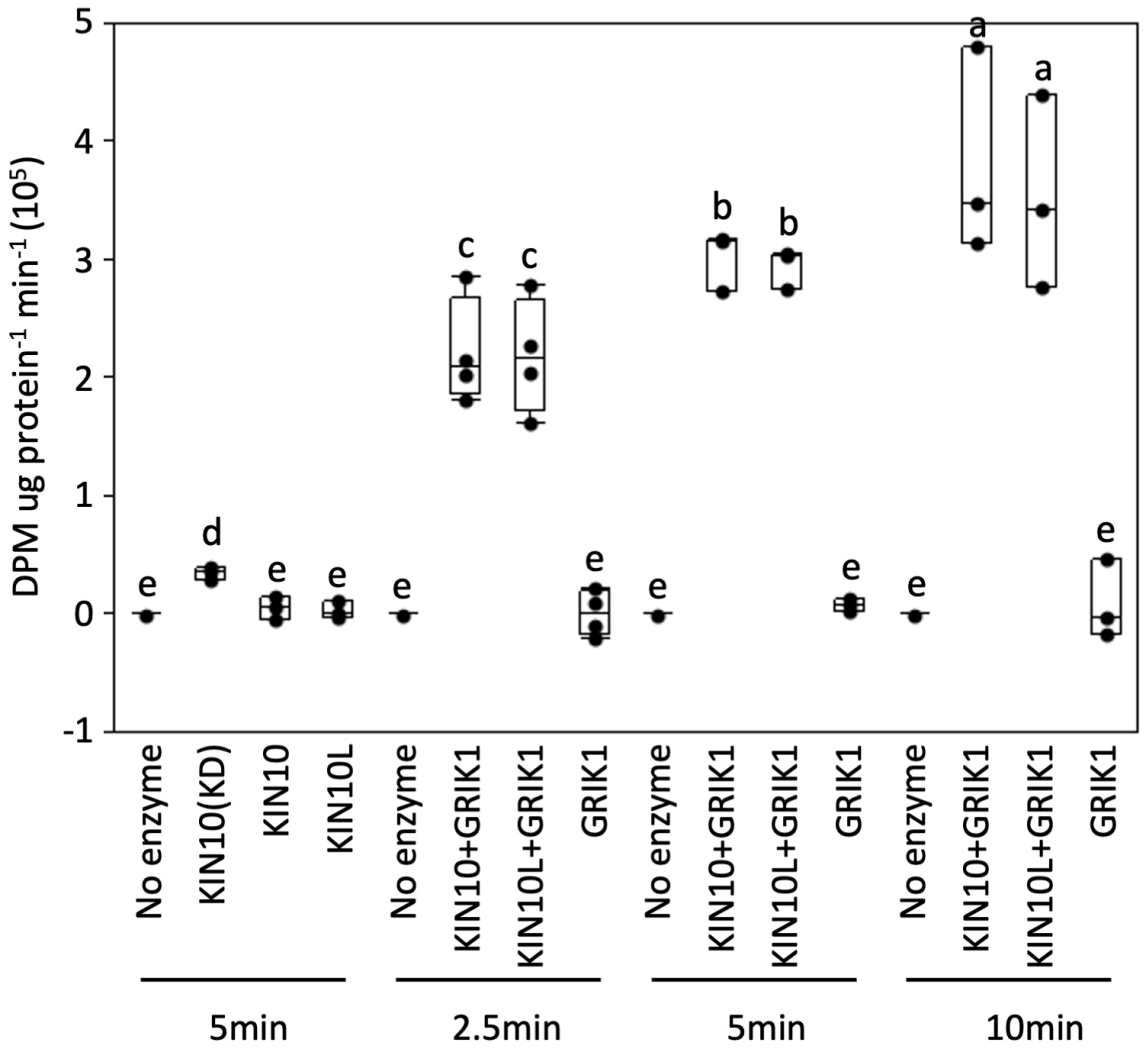
*In vitro* protein kinase assays of purified recombinant full length of KIN10 or KIN10L show that two KIN10 splicing isoforms have similar kinase activity. KIN10 activity is quantified by the incorporation of ^32^P from [γ-^32^P] ATP into the SPS peptide. Activity was measured in a 25µL-reaction containing the different KIN10 isoforms in the absence or presence of GRIK1 for the indicated times. KIN10 forms include KIN10(KD), the kinase domain of KIN10. KIN10, the short splicing isoform of KIN1. KIN10L, long splicing isoform of KIN10. The center line of the box and whisker plot denotes the mean, the box represents the interquartile range while the whiskers represent the 5th and 95th percentile (n = 3 or 4 independent biological replicates). One-way analysis of variance (ANOVA) and Tukey-Kramer Honestly Significant Difference (P <0.05) are used to compare means. Different letters above boxes indicate a significant difference.

Next, we tested whether the kinase activity of KIN10L is equivalent to that of KIN10 i.e., whether the extra 23AA at the N-terminus of KIN10L has any effect on its *in vitro* kinase assay. A His-trigger factor (TF) followed by a factor Xa protease cleavage site domain was fused to the N-termini of KIN10L or KIN10. The constructs were expressed in *E. coli* and the resulting protein products were purified with the use of Ni-NTA chromatography. KIN10L or KIN10 were recovered after factor Xa protease digestion to remove the affinity tag, yielding proteins with equivalent, i.e., approximately 95% purity as assessed by SDS-PAGE and Coomassie Brilliant blue staining (**Supplementary Figure 4**). For the kinase assays, a recombinant KIN10 kinase domain (KIN10KD) and GRIK1 were used as a positive kinase control and activator, respectively. The expected low i.e., basal levels of phosphorylation activity were observed in for KIN10L or KIN10 in the absence of GRIK1 activation, although KIN10KD showed higher activity than either isoform. Upon GRIK1 activation, the kinase activities of both KIN10L and KIN10 were elevated dramatically during a 10-minute time course during which no significant differences in kinase activity were detected between KIN10L and KIN10 (**Figure 5**).

## Discussion

SnRK1 is an evolutionarily conserved sensor kinase that plays critical roles in plant stress responses and development by regulating gene expression and enzyme activities. As a major kinase subunit of SnRK1, KIN10 is regulated by transcription, posttranscriptional modification, targeted protein degradation and subcellular localization. Among these regulatory mechanisms, targeted protein degradation of KIN10 is crucial for rapid SnRK1-regulated plant responses to ever-changing energy stress conditions. Since KIN10 kinase mutants such KIN10 (T175A) and KIN10 (K48A) were previously shown to be more stable and GRIKs are major kinases that activate KIN10, in this research we focused on the regulatory role of GRIK1 on KIN10 stability. The results from both protein transient expression assays in *N*. *benthamiana* leaves and the characterization of *grik* mutants supports the hypothesis that GRIK not only phosphorylates and activates KIN10 but also promotes its degradation. For GRIK1-mediated KIN10 degradation, we reason that the reduced stability of KIN10 upon phosphorylation and activation by GRIK represents a mechanism that enables the KIN10 activity to be rapidly reduced when the levels of intracellular sugar/energy are restored to their set point, representing an important level of homeostatic control that prevents a metabolic overreaction to low sugar conditions. Since GRIKs are activating kinases of KIN10, KIN10s in the *grik1 grik2* double null mutant background remain un-phosphorylated, with only their basal level of activity, are more stable, and therefore increase in cellular abundance, which also explains why the longer isoform KIN10L which is a minor isoform in wild type plant can be clearly detected in the *grik1 grik2* double mutant.

Several recent reports showed nuclear translocation of KIN10 plays an important regulatory contribution with respect to SnRK1 downstream regulation (Ramon et al., 2019; Belda-Palazón et al., 2022; Shi et al., 2022). For example, abscisic acid (ABA) exposure triggers rapid subcellular re-localization of KIN10 from the nucleus to the cytoplasm and this is accompanied by the inhibition of the target of rapamycin (TOR) sensor kinase (Belda-Palazón et al., 2022), implying that the subcellular re-localization of KIN10 likely exposes it to a different set of phosphorylation targets. In this study, characterizations of KIN10L and KIN10 show that although recombinant KIN10L and KIN10 demonstrate similar kinase activities with respect to *in vitro* kinase assay, KIN10L tends to accumulate to higher levels than KIN10 upon transient expression in *N*. *benthamiana*. Together with our observation that a putative subcellular localization mutant accumulates to different levels, implies that insufficient SnRK1 activity *in vivo* may activate a feedback mechanism to regulate the alternative splicing of *KIN10*. Future research on this feedback regulatory mechanism will likely provide additional insights into the complexity of SnRK1 signaling.

## Experimental procedures

### Plant materials and growth conditions

*grik* mutants: *grik1-2 grik2-1* and *grik1-1* (+/−) *grik2-1* (−/−) were obtained from Nathalie Glab (Institute of Plant Sciences Paris-Saclay, France) (Glab et al., 2017). Other mutants are used in this research including: *grik1-1* (CS2103211 or GK-713C09), *grik1-2* (SALK_142938), *grik2-1* (SALK_015230), *kin10-2* (SALK_093965). For experiments with the *grik1-1 grik2-1* double mutants, double homozygous null individuals *grik1-1(−/−) grik2-1(−/−)* were selected from the progeny of the *grik1-1(+/−) grik2-1(−/−)* sesqui parental line by genotyping with primers listed in **Supplementary Table S1**. For Arabidopsis, seeds were surface sterilized with 70% ethanol, then with 30% bleach (Clorox^®^) containing 0.01% Tween20 for 15min. Seeds were rinsed five times with sterile water before planted on the half strength of Murashige and Skoog (MS) medium supplemented with 1% of sucrose and 0.7% of agar. After stratified at 4°C in dark for 3 days, seeds were germinated and cultured in Percival^®^ plant tissue culture chamber. 10-day-old seedlings then were transplanted onto moist BM2 potting soil (Berger) and grown on the shelf of a walk-in growth chamber. Plants were grown with a light/dark cycle of 18h/6h at 23°C, photosynthetic photon flux density of 250 μmol m^−2^ s^−1^, and 75% relative humidity. *Nicotiana benthamiana* (*N*. *benthamiana seeds*) were directly germinated on BM2 potting soil (Berger). 4-week-old *N*. *benthamiana* plants were used for transient gene expression by agroinfiltration.

### Genetic constructs

KIN10 refers to AT3G01090. Coding sequences (CDS) of KIN10L, KIN10, GRIK1 were amplified by PCR from cDNAs using primers listed in **Supplementary Table S1**. CDS of KIN10L (T198A), KIN10L (K250A, K251A, K253A), GRIK (S261A), GRIK1 (K137A) and SV40-KIN10L-SV40 were generated by overlapping PCR with primers listed in **Supplementary Table S1**.

For expression in plants, the PCR products were cloned into the Invitrogen GATEWAY^M^ pDONR/Zeo vector (Thermo Fisher Scientific, Waltham, MA; www.thermofisher.com) using the BP reaction and sub-cloned (LR reaction) into the plant GATEWAY™ binary vector: pGWB414 (HA-tag at C terminal) or pMDC43 (GFP-tag at N terminal) (Nakagawa et al., 2007).

### Nicotiana benthamiana agroinfiltration

Transient gene expression in *N. benthamiana* by agroinfiltration was carried out according to a previous described procedure (Schütze et al., 2009). Leaves were harvested 3 days after agroinfiltration for imaging with a Leica SP5 confocal laser scanning microscope or protein content analysis.

RNA isolation and RT-qPCR were conducted according to the method described in (Zhai et al., 2021)with primers listed in **Supplementary Table S1**.

### Recombinant protein production and purification from *E. coli*

Recombinant KIN10L, KIN10, KIN10 kinase domain and GRIK1 proteins fused with N-terminal His-tag were expressed in *E. coli* BL21 (DE3). His-tagged protein purification was performed as previously reported (Nallamsetty and Waugh, 2007). For production and purification of KIN10L and KIN10, CDS of *KIN10L* and *KIN10* were cloned into pCold-TF (Takara Bio) for fusion with trigger factor (TF) between the *Nde*I and *Xba*I restriction sites. Subsequently, the TF was removed from fusion proteins in digestion with Xa factor protease.

### Antibody and immunoblotting

A total of 50 mg of freshly harvested leaves tissues were ground in liquid nitrogen and then mixed with 200 µL of preheated protein extraction buffer (8 M urea, 2% SDS, 0.1 M DTT, 20% glycerol, 0.1 M Tris-HCl, pH (6.8), and 0.004% Bromophenol Blue). After incubated at 80°C for 5min, Samples were centrifuged at 17,000 g before loading supernatants into SDS-PAGE (SurePAGE™, Bis-Tris, 4-20%, precast gel, Genscript). Primary antibodies: anti-KIN10 1:1000 (Catalog No. AS10919, Agrisera), anti-phosphorylated-KIN10/11 (Phospho-AMPK alpha-1,2 (Thr183, Thr172) Polyclonal Antibody) 1:1000 (Catalog No. PA5-17831, Invitrogen), anti-GFP 1:2000 (Catalog No. A6455, Invitrogen), and anti-HA 1:2000 (Catalog No. 71-5500, Invitrogen). Immunoblots of targeted proteins were visualized using HRP-conjugated secondary antibodies with 1:10000 dilution (Catalog No. AP187P, Millipore) with SuperSignal™ West Femto Maximum Sensitivity Substrate (Catalog No. 34095, ThermoFisher). Immunoblot signals were detected and digitalized with Image Quant LAS4000. Phos-tag SDS-PAGE was performed using Acrylamide-pendant phos-tag™ according to the manual of the Acrylamide-pendant Phos-tag™ kit obtained from Wako Chemicals (Richmond, VA). 50μM of Mn^2+^-phos-tag™ SDS-PAGE (10%) is to separate phosphorylated protein from its non-phosphorylated form.

### Kinase activity assay

For the KIN10 activity assay, 50 nM of purified KIN10 kinase domain (KD) or KIN10 or KIN10L or GRIK1 was diluted into 25-μL of Kinase Reaction Buffer containing 50 mM HEPES-NaOH, pH7.5, 5 mM MgCl2, 200 μM SPS peptide (RDHMPRIRSEMQIWSED), 4 mM DTT, 0.5 μM okadaic acid, 0.2 mM ATP, 12.2 kBq [γ-32P] ATP and incubated at 30°C for 5 min. The assay was stopped by transferring 10 μL of the assay mixture to 4-cm2 squares of Whatman P81 Phosphocellulose paper (Whatman, Maidstone, UK; www.gelifesciences.com/whatman), immersing it in 1% (v/v) phosphoric acid, then washing with four 800-mL volumes of 1% phosphoric acid. The paper squares were immersed in acetone, dried, and transferred to liquid scintillation vials to which 2 ml of Ultima Gold XR (PerkinElmer) was added before liquid scintillation counting using a Tri-carb (PerkinElmer) was used to determine radioactivity associated with phosphorylated SPS peptide (Zhai et al., 2018).

## Data availability statement

The original contributions presented in the study are included in the article/**Supplementary Material**. Further inquiries can be directed to the corresponding authors.

## Author contributions

JiS: Conceptualization, Formal analysis, Investigation, Writing – review & editing. HL: Formal analysis, Investigation, Writing – review & editing. JB: Formal analysis, Investigation, Writing – review & editing. YC: Formal analysis, Investigation, Writing – review & editing. ZZ: Conceptualization, Formal analysis, Writing – original draft. JoS: Conceptualization, Formal analysis, Funding acquisition, Writing – review & editing.

## Supplementary material

Supplementary Table 1 Oligonucleotide sequences of primers used in this study.

## References

[B1] Ananieva E. A. Gillaspy G. E. Ely A. Burnette R. N. Les Erickson F. (2008). Interaction of the WD40 domain of a myoinositol polyphosphate 5-phosphatase with SnRK1 links inositol, sugar, and stress signaling. Plant Physiol. 148, 1868–1882. doi: 10.1104/pp.108.130575 18931139 PMC2593651

[B2] Baena-González E. Lunn J. E. (2020). SnRK1 and trehalose 6-phosphate–two ancient pathways converge to regulate plant metabolism and growth. Curr. Opin. Plant Biol. 55, 52–59. doi: 10.1016/j.pbi.2020.01.010 32259743

[B3] Baena-González E. Rolland F. Thevelein J. M. Sheen J. (2007). A central integrator of transcription networks in plant stress and energy signalling. Nature 448, 938–942. doi: 10.1038/nature06069 17671505

[B4] Belda-Palazón B. Costa M. Beeckman T. Rolland F. Baena-González E. (2022). ABA represses TOR and root meristem activity through nuclear exit of the SnRK1 kinase. Proc. Natl. Acad. Sci. 119, e2204862119. doi: 10.1073/pnas.2204862119 35787039 PMC9282376

[B5] Bhalerao R. P. Salchert K. Bakó L. Ökrész L. Szabados L. Muranaka T.. (1999). Regulatory interaction of PRL1 WD protein with Arabidopsis SNF1-like protein kinases. Proc. Natl. Acad. Sci. 96, 5322–5327. doi: 10.1073/pnas.96.9.5322 10220464 PMC21862

[B6] Broeckx T. Hulsmans S. Rolland F. (2016). The plant energy sensor: evolutionary conservation and divergence of SnRK1 structure, regulation, and function. J. Exp. Bot. 67, 6215–6252. doi: 10.1093/jxb/erw416 27856705

[B7] Chelsky D. Ralph R. Jonak G. (1989). Sequence requirements for synthetic peptide-mediated translocation to the nucleus. Mol. Cell. Biol. 9, 2487–2492. doi: 10.1128/mcb.9.6.2487-2492.1989 2668735 PMC362321

[B8] Coello P. Hirano E. Hey S. J. Muttucumaru N. Martinez-Barajas E. Parry M. A.. (2012). Evidence that abscisic acid promotes degradation of SNF1-related protein kinase (SnRK) 1 in wheat and activation of a putative calcium-dependent SnRK2. J. Exp. Bot. 63, 913–924. doi: 10.1093/jxb/err320 21994172 PMC3254688

[B9] Crepin N. Rolland F. (2019). SnRK1 activation, signaling, and networking for energy homeostasis. Curr. Opin. Plant Biol. 51, 29–36. doi: 10.1016/j.pbi.2019.03.006 31030062

[B10] Crozet P. Jammes F. Valot B. Ambard-Bretteville F. Nessler S. Hodges M.. (2010). Cross-phosphorylation between Arabidopsis thaliana sucrose nonfermenting 1-related protein kinase 1 (AtSnRK1) and its activating kinase (AtSnAK) determines their catalytic activities. J. Biol. Chem. 285, 12071–12077. doi: 10.1074/jbc.M109.079194 20164192 PMC2852945

[B11] Crozet P. Margalha L. Butowt R. Fernandes N. Elias C. A. Orosa B.. (2016). SUMOylation represses SnRK1 signaling in Arabidopsis. Plant J. 85, 120–133. doi: 10.1111/tpj.13096 26662259 PMC4817235

[B12] Emanuelle S. Hossain M. I. Moller I. E. Pedersen H. L. Meene A. M. Doblin M. S.. (2015). SnRK1 from Arabidopsis thaliana is an atypical AMPK. Plant J. 82, 183–192. doi: 10.1111/tpj.12813 25736509

[B13] Figueroa C. M. Lunn J. E. (2016). A tale of two sugars: trehalose 6-phosphate and sucrose. Plant Physiol. 172, 7–27. doi: 10.1104/pp.16.00417 27482078 PMC5074632

[B14] Glab N. Oury C. Guerinier T. Domenichini S. Crozet P. Thomas M.. (2017). The impact of Arabidopsis thaliana SNF1-related-kinase 1 (SnRK1)-activating kinase 1 (SnAK1) and SnAK2 on SnRK1 phosphorylation status: characterization of a SnAK double mutant. Plant J. 89, 1031–1041. doi: 10.1111/tpj.13445 27943466

[B15] Han C. Liu Y. Shi W. Qiao Y. Wang L. Tian Y.. (2020). KIN10 promotes stomatal development through stabilization of the SPEECHLESS transcription factor. Nat. Commun. 11, 1–10. doi: 10.1038/s41467-020-18048-w 32843632 PMC7447634

[B16] Hey S. Mayerhofer H. Halford N. G. Dickinson J. R. (2007). DNA sequences from Arabidopsis, which encode protein kinases and function as upstream regulators of Snf1 in yeast. J. Biol. Chem. 282, 10472–10479. doi: 10.1074/jbc.M611244200 17237223

[B17] Jossier M. Bouly J. P. Meimoun P. Arjmand A. Lessard P. Hawley S.. (2009). SnRK1 (SNF1-related kinase 1) has a central role in sugar and ABA signalling in Arabidopsis thaliana. Plant J. 59, 316–328. doi: 10.1111/j.1365-313X.2009.03871.x 19302419

[B18] Lee J.-H. Terzaghi W. Gusmaroli G. Charron J.-B. F. Yoon H.-J. Chen H.. (2008). Characterization of Arabidopsis and rice DWD proteins and their roles as substrate receptors for CUL4-RING E3 ubiquitin ligases. Plant Cell 20, 152–167. doi: 10.1105/tpc.107.055418 18223036 PMC2254929

[B19] Lunn J. E. Feil R. Hendriks J. H. Gibon Y. Morcuende R. Osuna D.. (2006). Sugar-induced increases in trehalose 6-phosphate are correlated with redox activation of ADPglucose pyrophosphorylase and higher rates of starch synthesis in Arabidopsis thaliana. Biochem. J. 397, 139–148. doi: 10.1042/BJ20060083 16551270 PMC1479759

[B20] Mair A. Pedrotti L. Wurzinger B. Anrather D. Simeunovic A. Weiste C.. (2015). SnRK1-triggered switch of bZIP63 dimerization mediates the low-energy response in plants. Elife 4, e05828. doi: 10.7554/eLife.05828.043 26263501 PMC4558565

[B21] Margalha L. Confraria A. Baena-González E. (2019). SnRK1 and TOR: modulating growth–defense trade-offs in plant stress responses. J. Exp. Bot. 70, 2261–2274. doi: 10.1093/jxb/erz066 30793201

[B22] Nakagawa T. Suzuki T. Murata S. Nakamura S. Hino T. Maeo K.. (2007). Improved gateway binary vectors: High-performance vectors for creation of fusion constructs in Transgenic analysis of plants. Biosci. Biotechnol. Biochem. 71, 2095–2100. doi: 10.1271/bbb.70216 17690442

[B23] Nallamsetty S. Waugh D. S. (2007). A generic protocol for the expression and purification of recombinant proteins in Escherichia coli using a combinatorial His6-maltose binding protein fusion tag. Nat. Protoc. 2, 383–391. doi: 10.1038/nprot.2007.50 17406599

[B24] Peixoto B. Baena-González E. (2022). Management of plant central metabolism by SnRK1 protein kinases. J. Exp. Bot. 73, 7068–7082. doi: 10.1093/jxb/erac261 35708960 PMC9664233

[B25] Ramon M. Dang T. V. T. Broeckx T. Hulsmans S. Crepin N. Sheen J.. (2019). Default activation and nuclear translocation of the plant cellular energy sensor SnRK1 regulate metabolic stress responses and development. Plant Cell 31, 1614–1632. doi: 10.1105/tpc.18.00500 31123051 PMC6635846

[B26] Ramon M. Ruelens P. Li Y. Sheen J. Geuten K. Rolland F. (2013). The hybrid Four-CBS-Domain KIN βγ subunit functions as the canonical γ subunit of the plant energy sensor Sn RK 1. Plant J. 75, 11–25. doi: 10.1111/tpj.12192 23551663 PMC6599549

[B27] Schluepmann H. Pellny T. van Dijken A. Smeekens S. Paul M. (2003). Trehalose 6-phosphate is indispensable for carbohydrate utilization and growth in Arabidopsis thaliana. Proc. Natl. Acad. Sci. 100, 6849–6854. doi: 10.1073/pnas.1132018100 12748379 PMC164535

[B28] Schütze K. Harter K. Chaban C. (2009). Bimolecular fluorescence complementation (BiFC) to study protein-protein interactions in living plant cells. Plant Signal Transduct.: Methods Protoc., 189–202. doi: 10.1007/978-1-59745-289-2_12 19083187

[B29] Shen W. Hanley-Bowdoin L. (2006). Geminivirus infection up-regulates the expression of two Arabidopsis protein kinases related to yeast SNF1-and mammalian AMPK-activating kinases. Plant Physiol. 142, 1642–1655. doi: 10.1104/pp.106.088476 17041027 PMC1676070

[B30] Shen W. Reyes M. I. Hanley-Bowdoin L. (2009). Arabidopsis protein kinases GRIK1 and GRIK2 specifically activate SnRK1 by phosphorylating its activation loop. Plant Physiol. 150, 996–1005. doi: 10.1104/pp.108.132787 19339507 PMC2689985

[B31] Shi W. Wang L. Yao L. Hao W. Han C. Fan M.. (2022). Spatially patterned hydrogen peroxide orchestrates stomatal development in Arabidopsis. Nat. Commun. 13, 1–12. doi: 10.1038/s41467-022-32770-7 36028510 PMC9418256

[B32] Sugden C. Donaghy P. G. Halford N. G. Hardie D. G. (1999). Two SNF1-related protein kinases from spinach leaf phosphorylate and inactivate 3-hydroxy-3-methylglutaryl-coenzyme A reductase, nitrate reductase, and sucrose phosphate synthase in *vitro* . Plant Physiol. 120, 257–274. doi: 10.1104/pp.120.1.257 10318703 PMC59258

[B33] Tsai A. Y. L. Gazzarrini S. (2012). AKIN10 and FUSCA3 interact to control lateral organ development and phase transitions in Arabidopsis. Plant J. 69, 809–821. doi: 10.1111/j.1365-313X.2011.04832.x 22026387

[B34] Wang J. Guan H. Dong R. Liu C. Liu Q. Liu T.. (2019). Overexpression of maize sucrose non-fermenting-1-related protein kinase 1 genes, ZmSnRK1s, causes alteration in carbon metabolism and leaf senescence in Arabidopsis thaliana. Gene 691, 34–44. doi: 10.1016/j.gene.2018.12.039 30594634

[B35] Williams S. P. Rangarajan P. Donahue J. L. Hess J. E. Gillaspy G. E. (2014). Regulation of sucrose non-fermenting related kinase 1 genes in Arabidopsis thaliana. Front. Plant Sci. 5, 324. doi: 10.3389/fpls.2014.00324 25071807 PMC4090914

[B36] Yadav U. P. Ivakov A. Feil R. Duan G. Y. Walther D. Giavalisco P.. (2014). The sucrose–trehalose 6-phosphate (Tre6P) nexus: specificity and mechanisms of sucrose signalling by Tre6P. J. Exp. Bot. 65, 1051–1068. doi: 10.1093/jxb/ert457 24420566 PMC3935566

[B37] Zhai Z. Keereetaweep J. Liu H. Feil R. Lunn J. E. Shanklin J. (2018). Trehalose 6-phosphate positively regulates fatty acid synthesis by stabilizing WRINKLED1. Plant Cell. 30, 2616–2627. doi: 10.1105/tpc.18.00521 30249634 PMC6241258

[B38] Zhai Z. Keereetaweep J. Liu H. Feil R. Lunn J. E. Shanklin J. (2021). Expression of a bacterial trehalose-6-phosphate synthase otsA increases oil accumulation in plant seeds and vegetative tissues. Front. Plant Sci. 12. doi: 10.3389/fpls.2021.656962 PMC7988188 33777087

[B39] Zhai Z. Liu H. Shanklin J. (2017). Phosphorylation of WRINKLED1 by KIN10 results in its proteasomal degradation, providing a link between energy homeostasis and Lipid biosynthesis. Plant Cell 29, 871–889. doi: 10.1105/tpc.17.00019 28314829 PMC5435435

