# Supplementary material for: GRIK phosphorylates and activates KIN10 which also promotes its degradation
Source: Front Plant Sci. 2024 Mar 25;15:1375471. doi: 10.3389/fpls.2024.1375471 (PMC10999582; doi:10.3389/fpls.2024.1375471)
Supplement: Supplementary Table 1 — Oligonucleotide sequences of primers used in this study. [file Table_1.pdf]

TABLES

Table S1. Oligonucleotide sequences of primers used in this study.

Gene	Applications	Primer Pair Sequences (forward and reverse primer, 5'-3')
KIN10L	Expression in plant	GGGGACAAGTTTGTACAAAAAAGCAGGCTTC ATGGGAGATGGGAGTTCCAGTAG and GGGGACCACTTTGTACAAGAAAGCTGGGTCGAGGCGTCTG GATTTGTTAGG
KIN10L	Recombinan t protein	TCGAAGGTAGGCATATGATGTTCAAACGAGTAGATGAGTT T and AGATTACCTATCTAGATCAGAGGACTCGGAGCTGA
KIN10	Expression in plant	GGGGACAAGTTTGTACAAAAAAGCAGGCTTCATGTTCAAA CGAGTAGATGAGTTTAATT and GGGGACCACTTTGTACAAGAAAGCTGGGTCTCAGAGGACT CGGAGCTGAG
KIN10	Recombinan t protein	TCGAAGGTAGGCATATGATGGATGGATCAGGCACAG and AGATTACCTATCTAGATCAGAGGACTCGGAGCTGA
KIN10 kinase domain	Recombinan t protein	CTTGGAATTCATGGATGGATCAGGCACAGG and GAGTTCCTCGAGTCAATAACCACTAGAGGCACGGAAAC
GRIK1	Expression in plant	GGGGACAAGTTTGTACAAAAAAGCAGGCTTCATGTTTTGT GATAGTTTTGCATTTGCC and GGGGACCACTTTGTACAAGAAAGCTGGGTCGCTATGGTTTT GATCTTCTTCTTCTTCAG
GRIK1	Recombinan t protein	CTTGGAATTCATGTTTTGTGATAGTTTTGCA and GAGTTCCTCGAGTCAGCTATGGTTTTGATCTT
KIN10(T1 98A)	Expression in plant	GGACTTCCACAACTTGCCTTCAAAAAATGACCATCTCGC and GAAGGCAAGTTGTGGAAGTCCAAATTATGC
GRIK1(K1 37A)	Expression in plant	GACTTGTGAAAAGCCGCAATAGCATAATGCTTGTCATCGA CAG and CTATTGCGGCTTTTCACAAGTCACATTTATCG
GRIK1(S2 61A)	Expression in plant	CTTTGAATACTTGGGCGACACTGAAATCTCCTATTTTCACT C and GTGTCGCCCAAGTATTCAAAGATGATGATGATCAAC
KIN10 native promoter	Complemen tation	CCAAGCTTGCATGCCTTCACAGCTATACTATCTTCTCTTT and TGTTGATAACTCTAGGAGAATTTAGCGAGAATTAGGATCC
KIN10L(K 250A,K25 1A,K253A)	Expression in plant	CGCTATCGCCGCAAAAAGGTTGGGAATGTTTTCATCATC and CCCAACCTTTTTGCGGCGATAGCGGGAGGGATATACACATT ACCTAGCC
SV40NLS	Add SV40NLS to both N-terminus and C-terminus of KIN10L	ATGCCAAAGAAAAAGAGAAAGGTTTTCAAACGAGTAGATG AGTTT and TCAAACCTTTCTTTTCTTTTTAGGGAGGACTCGGAGCTGAG C GGGGACAAGTTTGTACAAAAAAGCAGGCTTCATGCCAAAG AAAAAGAGAAAGGTT and GGGGACCACTTTGTACAAGAAAGCTGGGTCTCAAACCTTTC TTTTCTTTTTAGG

KIN10	qPCR	AGAATGATGGCACTGTGACG and ACAGGTGAAGCAACGCTT
KIN11	qPCR	CCGTGTTCCAAGTGGCTATC and ACATGTGCAGGAATCCAGTG
F-box	qPCR	GGCTGAGAGGTTCGAGTGTT and GGCTGTTGCATGACTGAAGA
UBQ10	qPCR	ACCATCACTTTGGAGGTGGA and GTCAATGGTGTCGGAGCTTT
grik1-1(CS2103211)	genotyping	CAAACGCTTCTAATCGCCAAC and CATGGTGACATCAAACCTGAC
grik2-1 (salk_015230)	genotyping	TGCGGGATATTGTTACTGGAC and GTCCTGAAGAGTATCCGCAAG
