# Supplementary material for: GRIK phosphorylates and activates KIN10 which also promotes its degradation
Source: Front Plant Sci. 2024 Mar 25;15:1375471. doi: 10.3389/fpls.2024.1375471 (PMC10999582; doi:10.3389/fpls.2024.1375471)
Supplement: Supplementary file 2 [file DataSheet_1.pdf]

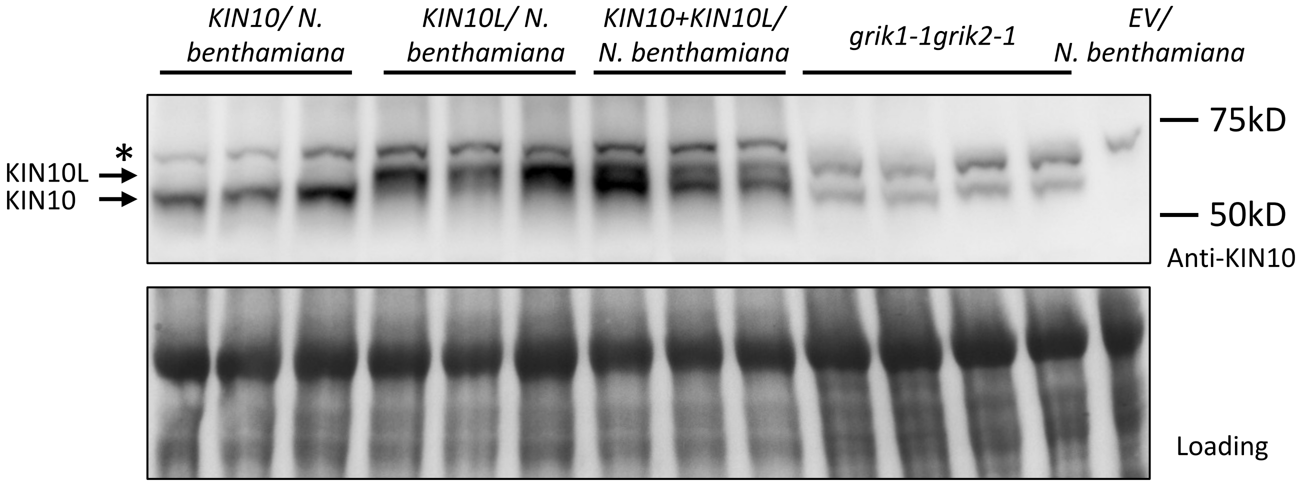

Supplemental Figure 1. Transient expressions of *KIN10L* and *KIN10* in *N. benthamiana* show their similar migrations to that of putative *KIN10* alternative splicing protein products in *grik1-1 grik2-1 mutant*. Immunoblot analysis of proteins extracted from either *N. benthamiana* leaves that were transiently transformed with either KIN10L or KIN10 or co-transformed with both respectively, and from 10-day-old *grik1-1 grik2-1* seedlings. Ponceau S staining of Rubisco is shown as a loading control.

Small band: TIC traces (Sciex 5600+ QqTOF with Eksigent uHPLC nano115)

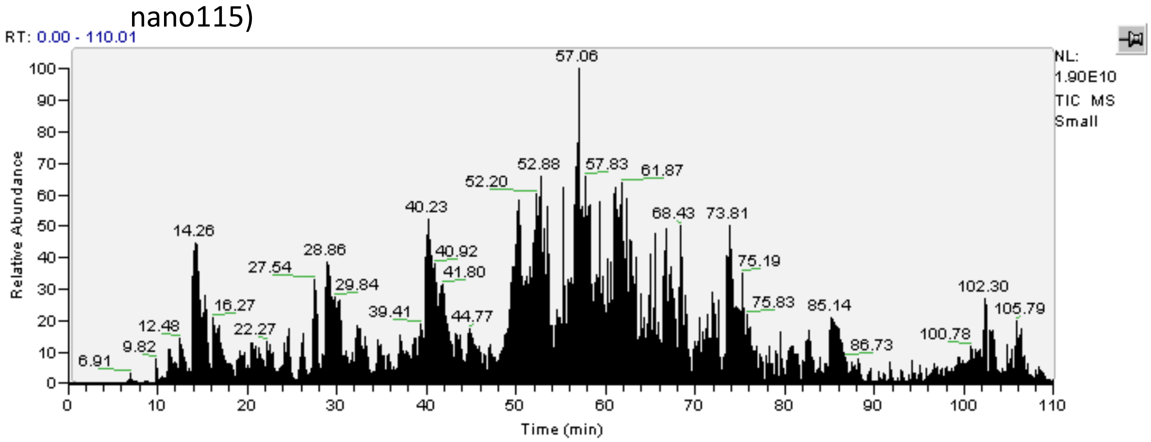

Identified as SNF1-related protein kinase catalytic subunit alpha KIN10(Q38997-2)

```
MDGSGTGSRSGVESILPNYKLGRTLGIGSFGRVKIAEHALTGHKVAIKILNRRKIKNMEMEEKVRRE
IKILRLFMHPHIIRLYEVIETPTDIYLVMEYVNSGELFDYIVEKGRLQEDEARNFFQQIISGVEYCH
RNMVVHRDLKPENLLLDSKCNVKIADFGLSNIMRDGHFLKTSCGSPNYAAPEVISGKLYAGPEVDVW
SCGVILYALLCGTLPFDDENIPNLFKKIKGGIYTLPSHLSPGARDLIPRMLVVDPMKRVTIPEIRQH
PWFQAHLPRYLAVPPPDTVQQAKKIDEEILQEVINMGFDRNHLIESLRNRTQNDGTVTYYLILDNRF
RASSGYLGAEFQETMEGTPRMHPAESVASPVSHRLPGLMEYQGVGLRSQYPVERKWALGLQSRAHPR
EIMTEVLKALQDLNVCWKKIGHYNMKCRWVPNSSADGMLSNSMHDNNYFGDESSIIENEAAVKSPNV
VKFEIQLYKTRDDKYLLDLQRVQGPQFLFLDLCAAFLAQLRVL
```

Big band: TIC traces

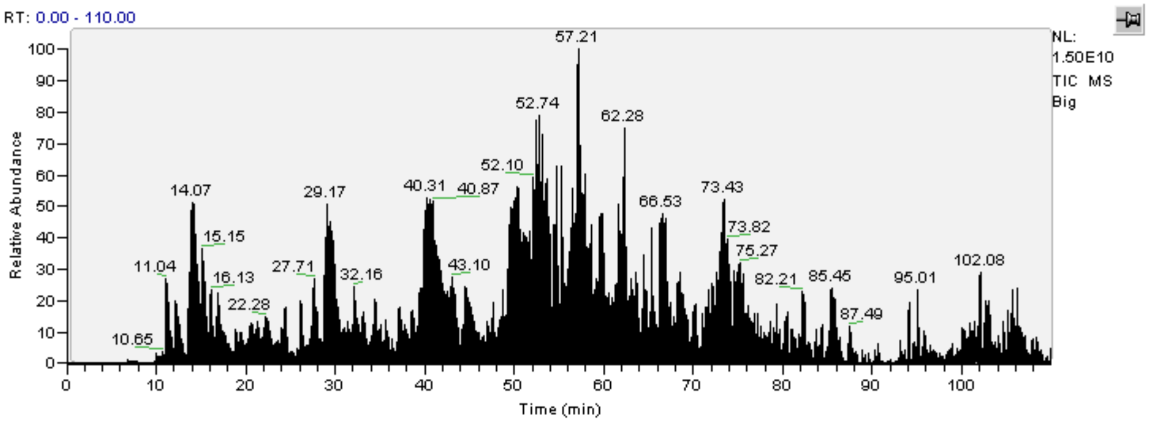

Identified as KIN10 Q38997-1 or Q38997-1)

```
MFKRVDEFNLVSSTIDHRIFKSRMDGSGTGSRSGVESILPNYKLGRTLGIGSFGRVKIAEHALTGHK
VAIKILNRRKIKNMEMEEKVRREIKILRLFMHPHIIRLYEVIETPTDIYLVMEYVNSGELFDYIVEK
GRLQEDEARNFFQQIISGVEYCHRNMVVHRDLKPENLLLDSKCNVKIADFGLSNIMRDGHFLKTSCG
SPNYAAPEVISGKLYAGPEVDVWSCGVILYALLCGTLPFDDENIPNLFKKIKGGIYTLPSHLSPGAR
DLIPRMLVVDPMKRVTIPEIRQHPWFQAHLPRYLAVPPPDTVQQAKKIDEEILQEVINMGFDRNHLI
ESLRNRTQNDGTVTYYLILDNRFRASSGYLGAEFQETMEGTPRMHPAESVASPVSHRLPGLMEYQGV
GLRSQYPVERKWALGLQSRAHPREIMTEVLKALQDLNVCWKKIGHYNMKCRWVPNSSADGMLSNSMH
DNNYFGDESSIIENEAAVKSPNVVKFEIQLYKTRDDKYLLDLQRVQGPQFLFLDLCAAFLAQLRVL
```

Supplemental Figure 2. Annotated MS/MS spectra showing both protein bands in the immunoblot assay for *grik1-1 grik2-1* (Figure 3) are identified as KIN10L and KIN10 respectively. KIN10 proteins were immunoprecipitated with KIN10 antibody from *grik1-1grik2-1* double mutant seedlings (10 days old), separated in SDS-PAGE gel and stained with Coomassie blue. Faster and slower migrating protein bands corresponding to those in the immunoblot were excised for protein identification with the use of tandem mass spectrometry (MS/MS). Amino acid sequences in red correspond to identified peptide fragments.

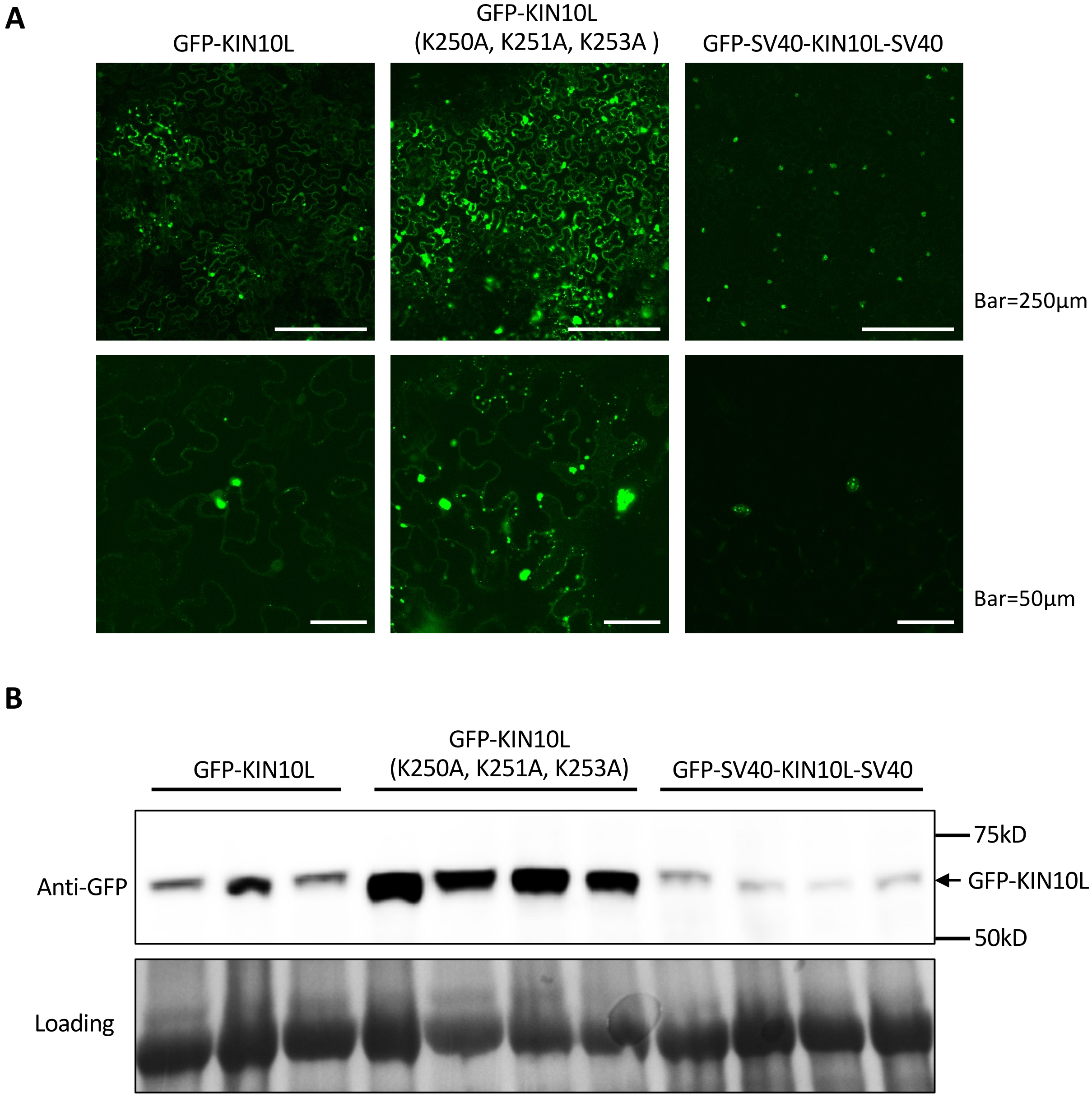

Supplemental Figure 3. A putative nuclear localization sequence (NLS) mutant of KIN10L is more stable, while a nuclear retained KIN10L is less stable than native KIN10L in *N. benthamiana* leaves transient assay. A putative NLS signal LFKKIKG in KIN10L matches perfectly with the proposed consensus sequence for monopartite NLSs-K·(K/R) ·X·(K/R) (Chelsky et al., 1989). To test this putative NLS, a mutant KIN10L (K250A, K251A, K253A) was generated and transiently expressed in *N. benthamiana*. Conversely, a nucleus targeted KIN10L (GFP-SV40-KIN10-SV40) in which SV40 NLS was fused to both N and C terminus of KIN10L was created and expressed in *N. benthamiana*. **A**, Representative fluorescence confocal images of *N. benthamiana* leaf samples 3 d after agroinfiltration with different gene as shown. **B**, immunoblot analysis of samples in (A) shows protein levels of GFP-KIN10L or GFP-KIN10L (K250A, K251A, K253A) or GFP-SV40-KIN10L-SV40. Ponceau S staining of Rubisco is shown as a loading control.

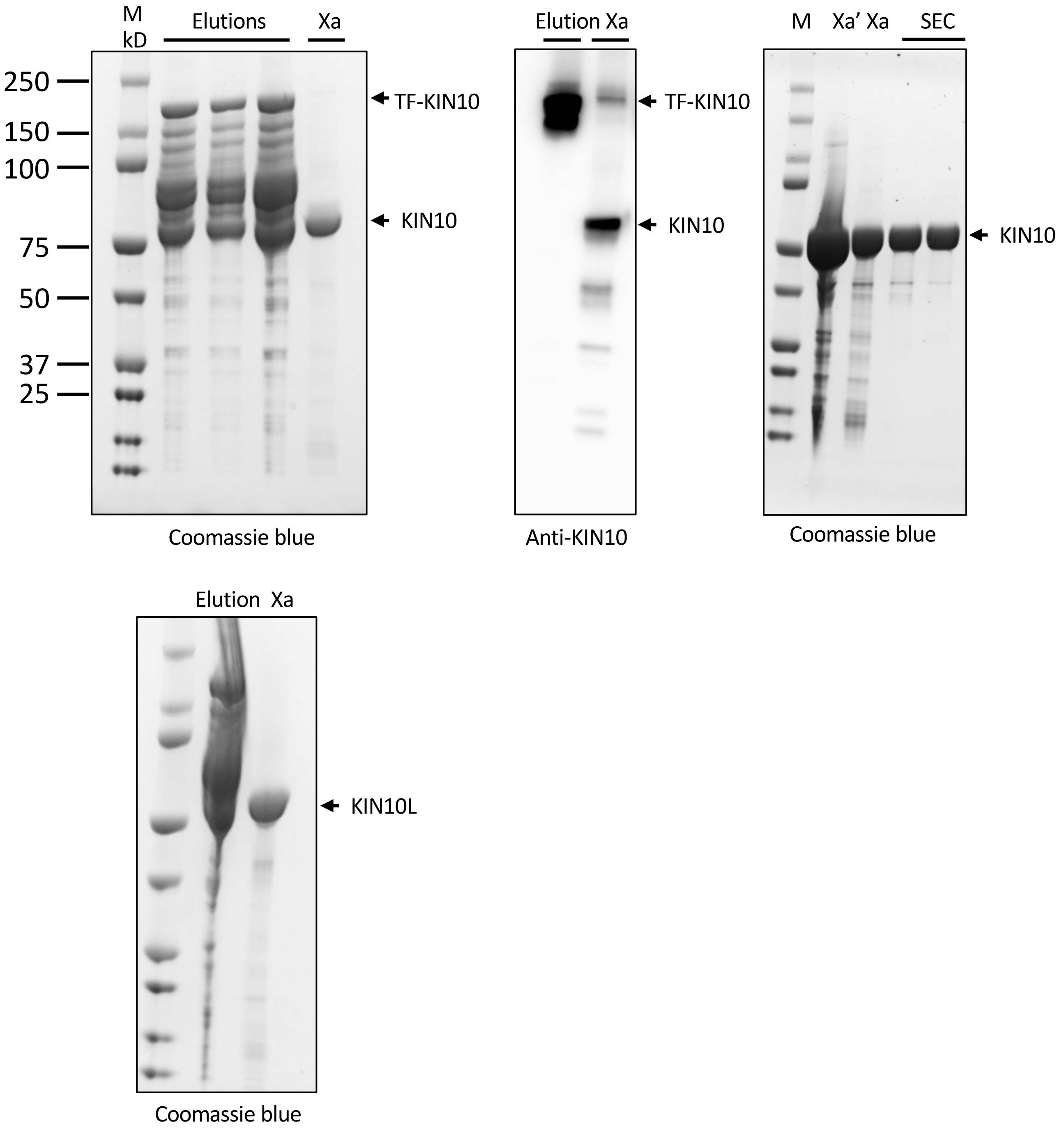

Supplemental Figure 4. Expression and purification of recombinant KIN10 (KIN10L and KIN10) from *E.coli*. Purified TF-KIN10 fusion proteins expressed from KIN10/pCold-TF (Elutions) were incubated with factor Xa protease on ice for 2 days (Xa') and re-purified by flowing through Ni-NTA (Xa) and further purification by Size Exclusion Chromatography (SEC). The identities of the resulting KIN10 proteins were confirmed by western blot using KIN10 antibody. KIN10L was produced and purified in the same way as for KIN10.

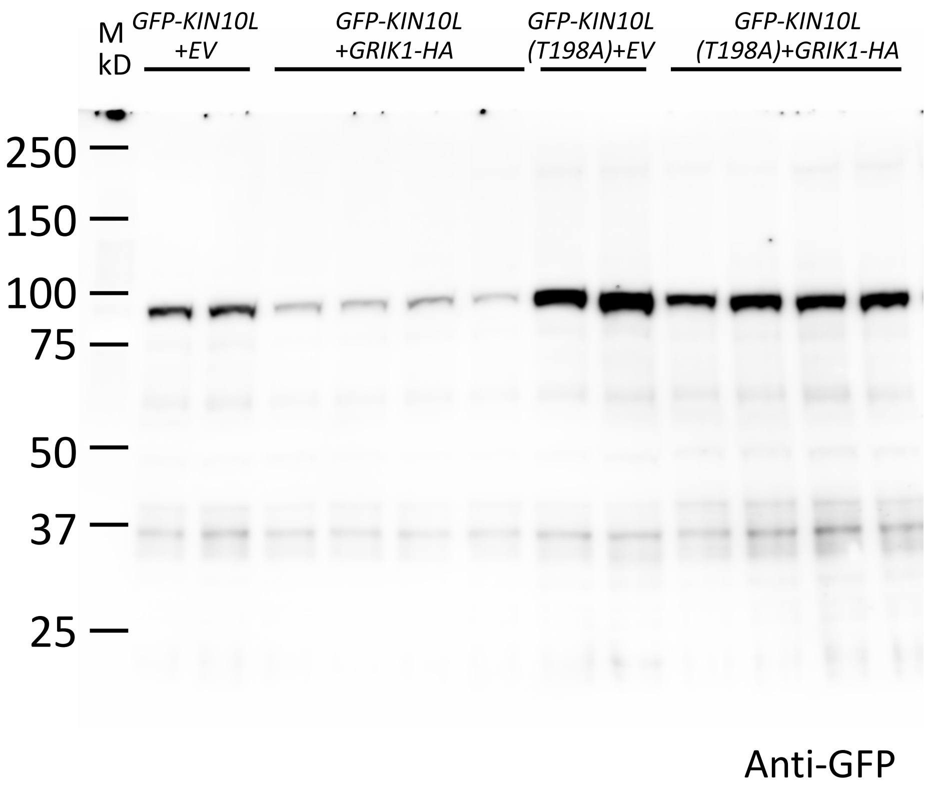

Supplemental Figure 5. A representative immunoblot for GFP-KIN10L in Figure 1B.

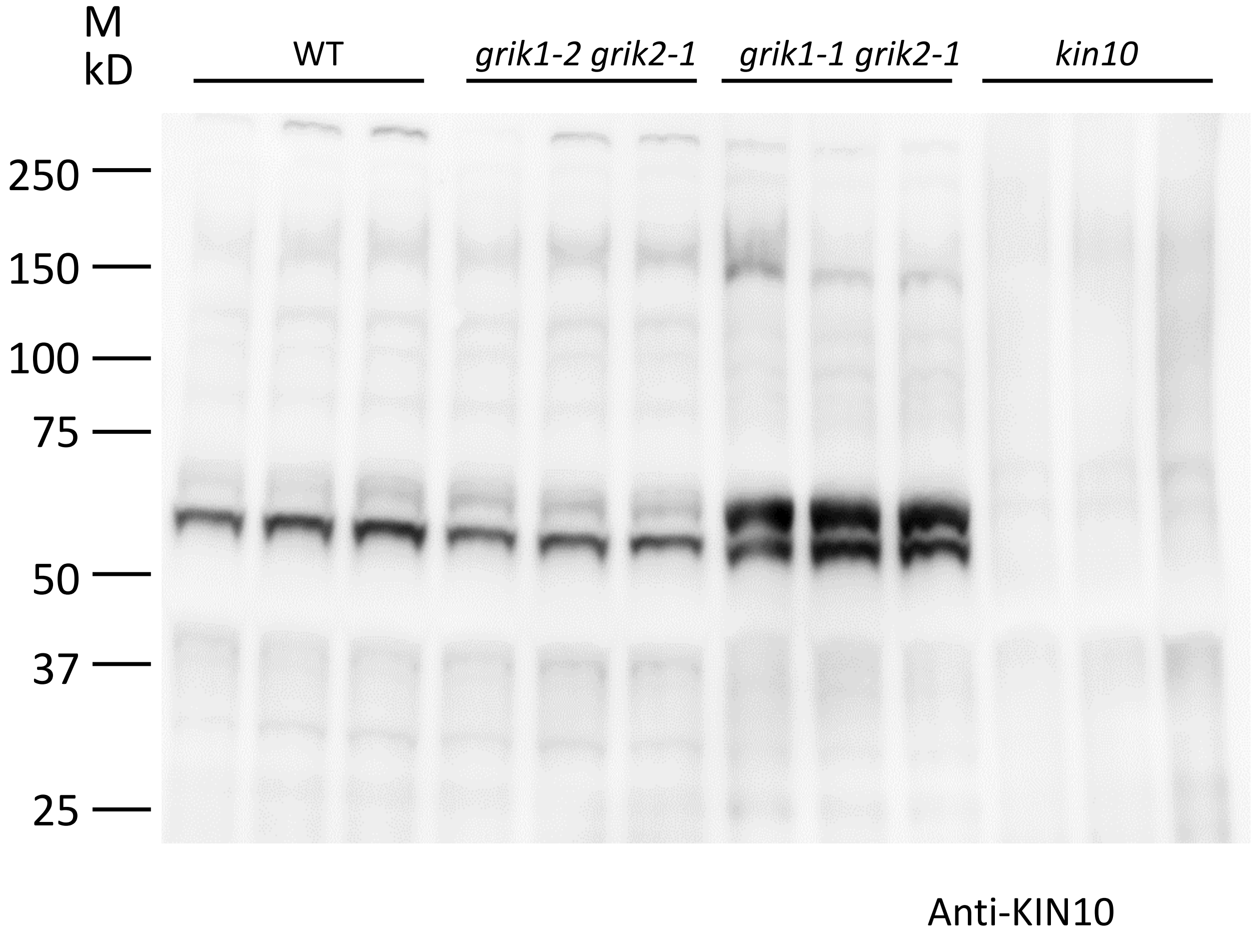

Supplemental Figure 6. A representative immunoblot for Figure 3A.
